# An integrative framework and recommendations for the study of DNA methylation in the context of race and ethnicity

**DOI:** 10.1007/s44155-023-00039-z

**Published:** 2023-04-20

**Authors:** Meingold Hiu-ming Chan, Sarah M. Merrill, Chaini Konwar, Michael S. Kobor

**Affiliations:** 1 Department of Medical Genetics, Faculty of Medicine, University of British Columbia, Vancouver, BC Canada; 2 British Columbia Children’s Hospital Research Institute, University of British Columbia, Vancouver, BC Canada; 3 Centre for Molecular Medicine and Therapeutics, University of British Columbia, Vancouver, BC Canada; 4 Edwin S. H. Leong Healthy Aging Program, Faculty of Medicine, University of British Columbia, Vancouver, BC Canada

## Abstract

Human social epigenomics research is critical to elucidate the intersection of social and genetic influences underlying racial and ethnic differences in health and development. However, this field faces major challenges in both methodology and interpretation with regard to disentangling confounded social and biological aspects of race and ethnicity. To address these challenges, we discuss how these constructs have been approached in the past and how to move forward in studying DNA methylation (DNAm), one of the best-characterized epigenetic marks in humans, in a responsible and appropriately nuanced manner. We highlight self-reported racial and ethnic identity as the primary measure in this field, and discuss its implications in DNAm research. Racial and ethnic identity reflects the biological embedding of an individual’s sociocultural experience and environmental exposures in combination with the underlying genetic architecture of the human population (i.e., genetic ancestry). Our integrative framework demonstrates how to examine DNAm in the context of race and ethnicity, while considering both intrinsic factors—including genetic ancestry—and extrinsic factors—including structural and sociocultural environment and developmental niches—when focusing on early-life experience. We reviewed DNAm research in relation to health disparities given its relevance to race and ethnicity as social constructs. Here, we provide recommendations for the study of DNAm addressing racial and ethnic differences, such as explicitly acknowledging the self-reported nature of racial and ethnic identity, empirically examining the effects of genetic variants and accounting for genetic ancestry, and investigating race-related and culturally regulated environmental exposures and experiences.

**Supplementary Information:**

The online version contains supplementary material available at 10.1007/s44155-023-00039-z.

## Introduction

The burgeoning field of human social epigenomics is a critical avenue for studying how the external social environment can get “under the skin” and influence key biological systems, a process known as biological embedding, through gene regulation with implications for human health and development [1–4]. For example, research efforts have been devoted to unraveling the molecular mechanisms underlying the extensively documented health disparities across race and ethnicity, especially in the USA. In particular, there is increasing interest in DNA methylation (DNAm), a commonly interrogated epigenetic mark in human population studies, to explain racial and ethnic health disparities [5, 6]. However, by examining the intersection of genetic and environmental influences, this field has encountered an issue vital for both methodology and interpretation: how to disentangle the confounded social and biological aspects of constructs, such as race and ethnicity.

The field of epigenetics, like many other areas of biomedical research, has historically used race and ethnicity to categorize study participants [7]. However, both concepts are increasingly recognized not as biologically meaningful categories [8, 9] but as complex sociocultural constructs that may reflect the biological embedding or embodiment of an individual’s social experience and environmental exposures in combination with their underlying genetic architecture. Given the history and inherent dangers of the misuse of biomedical science, including epigenomics, to reinforce racial stereotypes, robust investigations and careful interpretation of results related to race and ethnicity are required to avoid unintended consequences.

Building on progress in this field to date, this review discusses how race and ethnicity have been approached in the past and how to move forward taking account of these constructs in epigenetics research in a responsible and appropriately nuanced manner. We propose an integrative framework for studying DNAm in the context of race and ethnicity. This framework highlights and integrates specific aspects of both biological and environmental factors relevant to race and ethnicity in DNAm research, which can also be applied to other omics research. Given the interest in using DNAm to understand health disparities across race and ethnicity, we review the current state of research in this field based on this framework and discuss challenges and limitations in the field. We provide recommendations for conducting responsible and methodologically robust research in both the short and long term. Although this review is centered on DNAm, the overall framework can also be expanded and applied to other omics research, including gene expression and proteomics studies [3].

## Race and ethnicity measures in biomedical research

Race and ethnicity are both social constructs which, while commonly considered synonymous, represent distinct concepts. Specifically, race is commonly defined by physical traits, including skin color and hair texture, while ethnicity is a more complex and multidimensional construct reflecting cultural and historical background, including shared language, norms, traditions, values, diet, and geographical origin [10, 11]. Both have long been used in biomedical research as proxies for genetic population differences, especially as genotype data are not always available, which could contribute to the propagation of harmful racial stereotypes [12]. However, as clearly illustrated in the literature, different racial and ethnic groups are not genetically discrete [11], with more genetic variation within than across groups (93%–95% vs. 3%–5%, respectively) [13]. Further, these groupings based on race and ethnicity may be neither clinically meaningful nor appropriate. For example, in a study examining drug responses of individuals from eight different ethnic groups, four clusters inferred from genetic markers did not necessarily correspond to the groupings based on ethnicity [14].

Importantly, both race and ethnicity are commonly assessed by self-identification. At times, these constructs are inferred by a third party, such as health care providers, without soliciting self-reporting [15], or no details are provided regarding how they were measured [16]. However, as biomedical studies have often assessed race and ethnicity based on self-reporting [10, 17], we use the term “racial and ethnic *identity*” throughout this review where appropriate. Very few studies relied on other measures reflecting social standing (e.g., other-perceived racial or ethnic groups) or cultural orientations. Often, these measures are assessed using forced multiple choice questions, which can also impact the accuracy of reporting in medical research settings depending on the choices available and whether multiple identities can be selected [18]. Historically, participants who do not clearly fit into available categories may choose “other” as an option, and are subsequently grouped together with many unrelated identities or may be excluded from the analysis altogether [19]. In 2000, the U.S. Census updated their race and ethnicity measures to allow selection of multiple options, and thus documented that 2.4% of the U.S. population self-identified as multiracial. The 2020 U.S. Census revealed a 276% increase in the number of individuals identified as multiracial from 9 to 33 million since 2010, illustrating the increasing racial diversity of the U.S. population [20]. This further reinforced the importance of clearly stating exactly what was measured, i.e., racial and ethnic identity, along with the way in which it was measured to allow appropriate interpretation.

Racial and ethnic identity is a powerful determinant of social experience and environmental exposures. In countries where such identity is associated with social standing and underscores health outcomes, these constructs are likely to capture experiences of social inequalities and structural disparities rather than reflecting true biological differences [16]. Measures of race and ethnicity in health research are more common in North America, and less so in Europe and Asia [11, 16]. Given the lack of data, therefore, health disparities across race and ethnicity may remain undocumented in some countries.

## DNA methylation to explain racial and ethnic differences

Epigenetics, which sits at the interface of genes and the environment, offers a promising avenue to scrutinize how social and genetic factors together contribute to differential epigenetic signals across race and ethnicity. Specifically, DNAm has been increasingly appreciated and studied as a molecular mark pertinent to understanding racial and ethnic differences [5]. Typically, DNAm refers to the attachment of a methyl group to cytosine residues in the genome, especially at cytosine-phosphate-guanine (CpG) dinucleotides [21]. Similar to many other epigenetic modifications, DNAm has the potential to alter gene expression without changing the underlying DNA sequence. There is now strong evidence that the environment can contribute to differential DNAm, especially from studies in monozygotic twins with different environmental exposures exhibiting differences in epigenetic signatures, including DNAm and histone modifications [22]. While the relative impacts of genetics and environment are still under investigation, it is well established that DNAm is highly susceptible to the influences of both factors [23]. This malleability along with its mitotic heritability and stability over cellular generations make DNAm an ideal candidate for studying biological embedding, especially in the context of constructs such as race and ethnicity, which are simultaneously influenced by differences in genetics and sociostructural environments [21, 24].

Variations in DNAm have been explored to explain phenotypic differences across racial and ethnic groups, especially in the context of health disparities [6]. Substantial differences in a wide range of health risks and outcomes across race and ethnicity have long been recognized in North America, including disparities in rates of cancer [25, 26], chronic illnesses such as obesity [27], cardiovascular disease [28, 29], birth outcomes [30], and neurocognitive [31] and mental health conditions [32], and more recently infection and mortality associated with COVID-19 [33–35]. In addition to identifying health conditions with such racial and ethnic disparities, a growing body of research has attempted to elucidate the underlying factors and biological mechanisms [8]. Although much early research in the biomedical field assumed that genetic variants could explain health disparities across racial and ethnic identities, the recent discovery of a downsized human genome and the recurring issue of missing heritability suggested the importance of accounting for environmental effects [34, 36]. The global COVID-19 pandemic has further illustrated the need to understand health disparities through the lens of social determinants of health and to consider how societal contexts, such as living conditions, population density, social interactions, and access to/interactions with health care, can interact with processes within the body (e.g., immune response), which may exacerbate the risks of infection and mortality [34]. The need to expand research on social determinants of health and move beyond treating race and ethnicity solely as demographic characteristics has been strongly emphasized in social epidemiology and also discussed in the fields of social epigenomics [37] and other biomedical areas [38–40]. Given that health disparities are defined as “systematic, avoidable and unfair differences in health outcomes that can be observed between populations, between social groups within the same population or as a gradient across a population ranked by social position” (p. 28) [41], it is especially critical to examine how known sociostructural pressures may explain health disparities and influence DNAm in conjunction with other biological factors. In addition, the Developmental Origins of Health and Disease (DOHaD) hypothesis further posited that health disparities may originate from prenatal and early-life exposures, at times with racial underpinnings [42], resulting in predisposition of an individual to increased risk of developing diseases and poorer health outcomes over their life course [43]. A recent review identified a total of 49 epigenetics-related studies addressing racial and ethnic health disparities, with the majority focusing on DNAm, and documented increasing interest in this research area over the past several years [6]. Despite the burgeoning interest in this area, there has been limited discussion regarding how to examine DNAm in the context of racial and ethnic identity and related social constructs in a robust and nuanced manner while also considering the genetic backbone upon which DNAm is built [11].

## The current review

Building on prior efforts [6, 11, 44], this review continues the ongoing discussion of how to study DNAm in relation to race and ethnicity in a methodologically robust and culturally sensitive manner. We first discuss how both genetic ancestry and structural/sociocultural factors are confounded with racial and ethnic identity in a general context. Second, we introduce an integrative framework with a DOHaD and sociocultural perspective to guide future DNAm research in the context of race and ethnicity considering both genetic and environmental factors along with other biological influences relevant to DNAm. Based on this framework, we review the current state of DNAm studies addressing racial and ethnic health disparities, as this is a highly active and relevant field concerning racial and ethnic differences in DNAm, to identify existing limitations and present recommendations for future research in this field.

## Interrelations of racial and ethnic identity, genetic ancestry, and sociocultural experience/environment

**Fig. 1:**
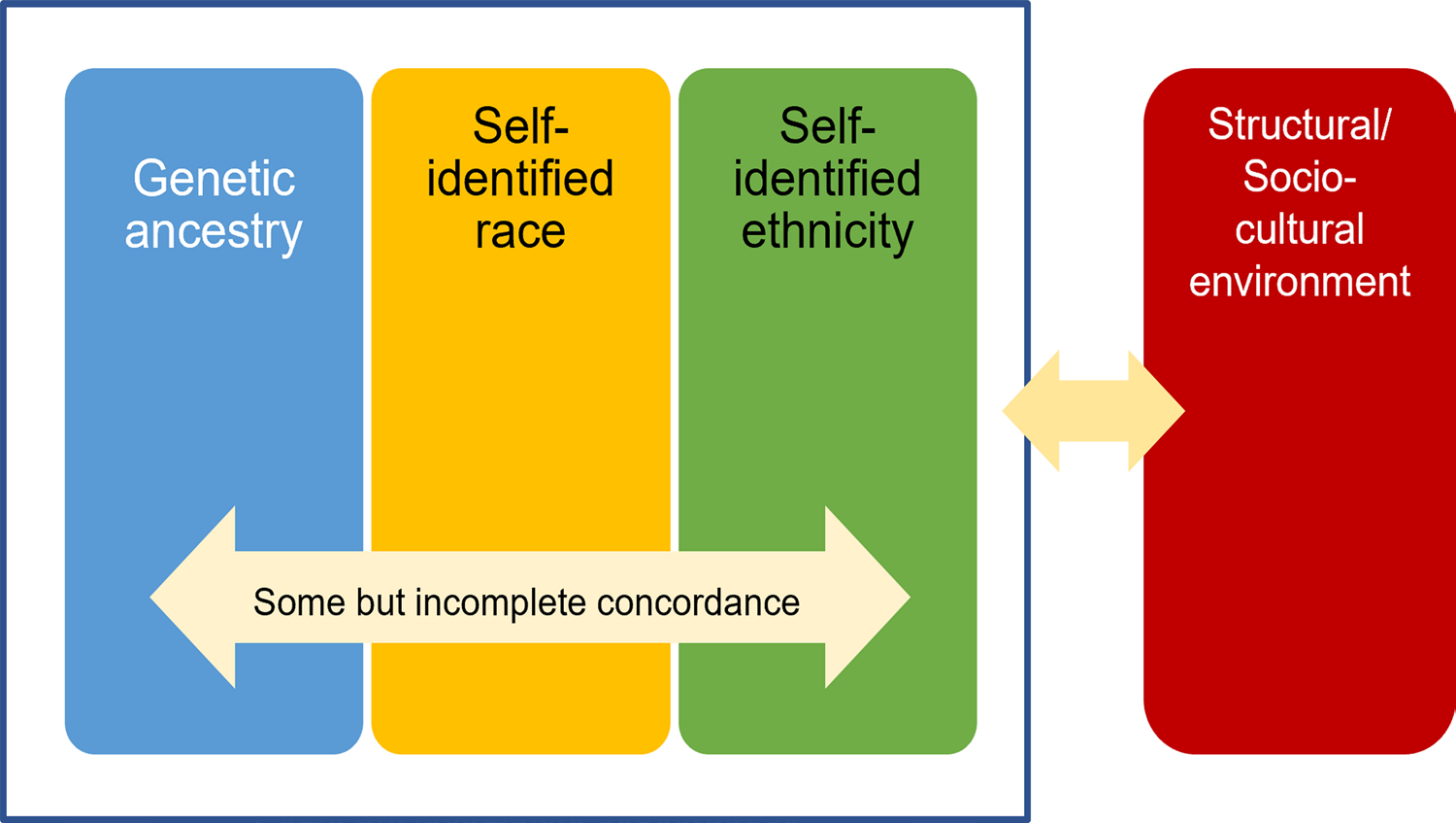
Interrelations between genetic ancestry, self-identified race and ethnicity, and structural/sociocultural environment. The first three constructs (blue box), which interact with the structural and sociocultural environment, have some albeit incomplete concordance and are at times used as proxies for each other. Figure modified from Lu et al. [16]

In the context of biomedical research, it is crucial to acknowledge that race and ethnicity are typically self-identified measures, which are inherently confounded with two interrelated components: genetic ancestry and social experience/environmental exposure (Fig. 1).

An individual’s racial and ethnic identity is an internal construct determined by the complex interplay of extrinsic and intrinsic factors. Extrinsic factors, such as structural and sociocultural environmental factors, may have bidirectional relations with racial and ethnic identity—the environment in which one is raised can contribute to the development of identity, which can also be a powerful determinant of one’s sociocultural environment and experience. As discussed previously, race and ethnicity as sociopolitical constructs can vary with time and their interpersonal and political contexts [45–48], such as immigration and political narratives around certain racial or ethnic groups. At the same time, an individual’s own identification with particular racial and ethnic groups can also change over time, sometimes in reaction to changing political narratives and/or along with the process of identity development and feelings of group belonging. This is especially true for multiracial individuals, who are increasingly prevalent in the population. Even the recent increase in availability of genetic testing from commercial entities, such as *23 and Me* and *Ancestry.com*, has also introduced knowledge of one’s genetic ancestry as a factor that may influence racial and ethnic identity, including the acceptance of these results [49]. In turn, the process of racial and ethnic identity development can influence an individual’s perception of their contexts and subsequent potential positive and negative health impacts [45, 50, 51]. Therefore, given their sociocultural and structural contexts as well as their effects on environmental exposures, it is necessary to take identity development into consideration when interpreting any racial or ethnic differences, ideally through empirical investigation. In this review, we specifically focus on structural factors, such as racism and socioeconomic disadvantage, and culturally regulated sociocultural factors, such as cultural norms and customs.

Racial and ethnic identity is often used as a proxy of genetic relatedness, and specifically genetic ancestry, a biological construct capturing the differences in genetic architecture described by the ancestral population structure. Genetic ancestry has some, albeit incomplete, concordance with racial and ethnic identity; individuals with the same racial or ethnic identity can have different genetic ancestries, and conversely those with the same genetic ancestry can identify as belonging to different racial or ethnic groups [15]. Indeed, studies have shown that racial and ethnic identity does not always accurately reflect genetic ancestry [10, 14].

While the importance of genetic ancestry in explaining DNAm variation is now increasingly appreciated among researchers, a common understanding of this construct is derived from the continent of origin concept. Continental-level ancestry groupings have been shown to capture sizable population differences in human genetic variation corresponding to geographical regions or superpopulations largely representing the major continents of the world [52, 53]. However, these continental-level ancestry categories are often confounded with racial or ethnic identities, which therefore challenges their applicability. Furthermore, such clustering overlooks genetic diversity within a continent, which is inaccurate given that the highest degree of genetic variation is observed within Africa [54, 55].

In addition, continental-level ancestry groupings neglect global and continuous patterns of migration along with the resulting extent of admixture across superpopulations and continents, which is misleading. For example, modern Latin Americans represent a classic admixed group comprised of African, Native American, and European ancestral parent genomes [56]. In admixed individuals, the fraction of genomic ancestry unique to the ancestral source populations can be estimated using global genetic ancestry methods [57, 58]. Alternatively, estimates of genetic ancestry for each of the chromosomes can be inferred by locus-specific local genetic ancestry measures. Therefore, genetic ancestry has emerged as an appropriate measure to capture differences in human genetic variation, which is best described along a continuum rather than in discrete categories, and thus explicitly captures the impacts of human histories that have led to different forms of genetic diversity around the globe.

## An integrative framework to study DNAm in the context of race and ethnicity

**Fig. 2:**
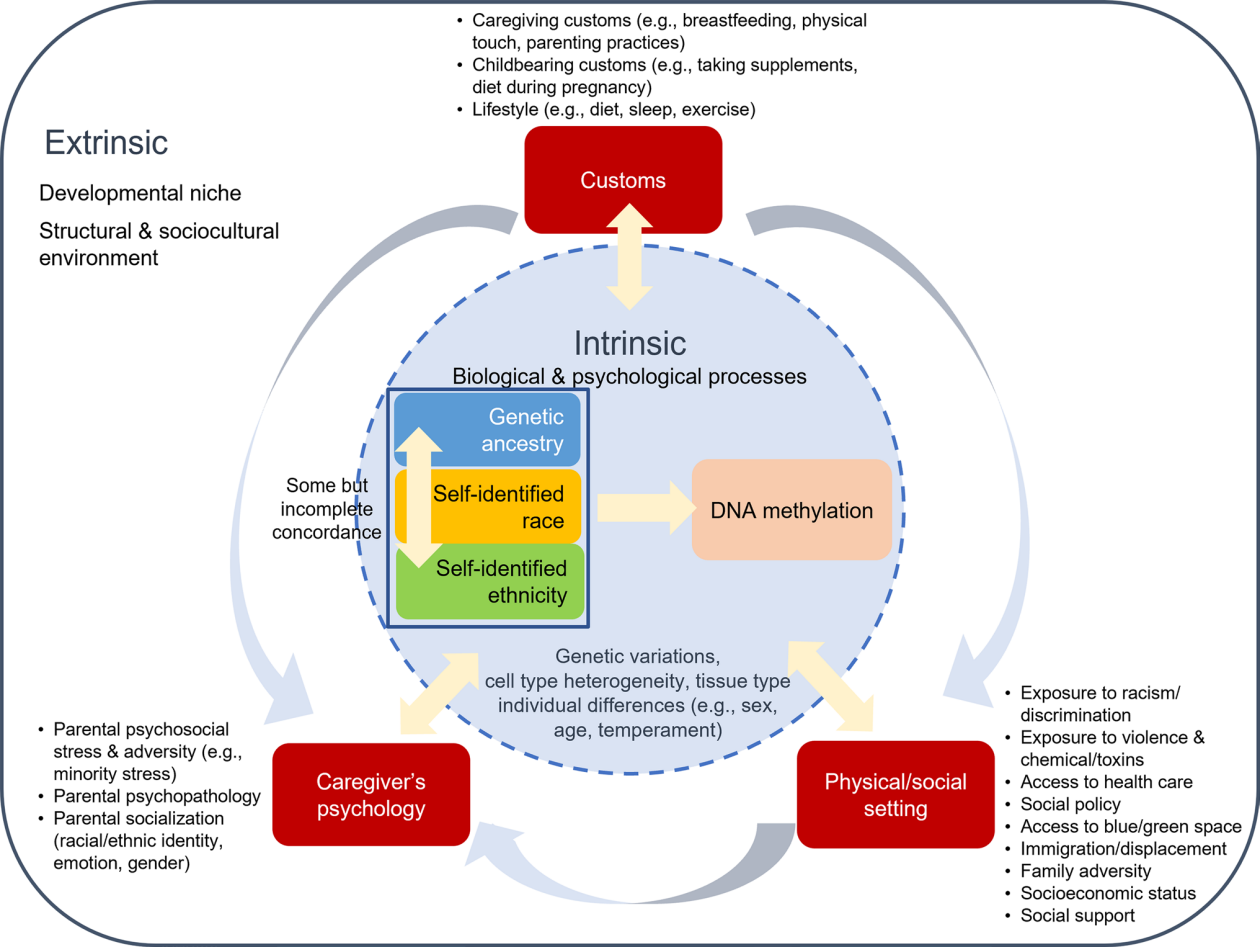
Integrative framework illustrating how intrinsic and extrinsic environmental factors tied to self-identified race and ethnicity contribute to DNA methylation (DNAm), which has the potential to alter gene expression and influence downstream biological functions associated with health and diseases. The circle represents an individual, with all the components inside the circle as intrinsic factors. The three main constructs—genetic ancestry, self-identified race, and self-identified ethnicity—are highlighted by the colored boxes, with other intrinsic factors that may influence DNAm encompassed within the circle. The extrinsic part is made up of both developmental niche and other structural and sociocultural environmental factors shown in red boxes. The developmental niche surrounding the individual consists of three subsystems, i.e., physical/social settings, customs, and caregiver’s psychology, which bring culture into their daily experience. Specific factors under each of the three subsystems of the developmental niche associated with DNAm and self-identified race and ethnicity are listed. The arrows between the three subsystems as well as those between the extrinsic and intrinsic factors represent their constant interactions and their contributions to the relations of race, ethnicity, and genetic ancestry with DNAm

To facilitate nuanced, precise, and responsible social epigenomics studies, we propose an integrative framework to guide the investigation of DNAm in the context of race and ethnicity (Fig. 2). This framework outlines how genetic ancestry and structural/sociocultural environment are both linked to self-identified racial and ethnic identity, together contributing to DNAm. Echoing the agenda of conceptualizing human biology as social biology [34], we propose a framework integrating both intrinsic and extrinsic factors potentially underlying the relations of DNAm with race and ethnicity. These factors are interdependent and interact to connect elements within and outside the body [34]. To specify our conceptualization of the extrinsic environment surrounding individuals in early childhood and over the life course, we adopted the developmental niche model, a well-established cultural research framework for child development [59, 60].

### Developmental Niche

The developmental niche model is an influential framework in the field of developmental science and cultural anthropology [59], and has been instrumental in guiding developmental research to study the cultural regulation of children’s immediate environment responsible for shaping their long-term development. Another significant contribution of this model is its emphasis on the “immediacy of cultural forces in the environment of an individual” (p. 284) [61], which helps to conceptualize and operationalize culture as a factor with direct impacts on the construction of children’s everyday experience [59]. Adopting the developmental niche model allows us to examine extrinsic early-life factors that may explain some of the epigenetic signals found in relation to race and ethnicity, such as signals found in certain racial groups but not others, in a systematic manner. This model also incorporates psychobiological concepts, such as child temperament and stress regulation, and highlights the interaction between children’s intrinsic biological factors and extrinsic cultural factors, which can be meaningfully adopted in the study of DNAm.

### Intrinsic factors

The operational definition of intrinsic factors for our framework includes those aspects arising from the self, both biological and psychological, for which there is evidence of associations with DNAm. Genetic ancestry and other biological factors, such as single nucleotide polymorphisms (SNPs) defined as genetic variations at the individual base pair level, tissue type, cell type heterogeneity, sex, and age, are all known to be drivers of variable and differential DNAm [24, 62]. As discussed above, self-identified race and ethnicity, referring to the internal psychological representation of two distinct but related group memberships, share some, albeit incomplete, concordance with genetic ancestry [14]. Other psychological traits, such as temperament (i.e., predisposed patterns of emotional and behavioral responses), are also associated with health outcomes and DNAm.

Many of these intrinsic factors simultaneously contribute to DNAm and shape the extrinsic environment. For example, psychological traits, such as temperament, are determined by genetics and can shape an individual’s susceptibility to their environment. Children with a fearful temperament are more likely to develop psychopathology when exposed to a stressful environment than their less fearful counterparts [63]. In addition, genetics, specifically the sex chromosomes, is associated with an individual’s sex, which also informs their gender identity and determines their social experience based on cultural expectations of gender roles. Similarly, social experiences change over the life course associated with cellular aging (which can be captured by epigenetic aging), changes in cell type composition, and health status. Therefore, it is necessary to recognize the complex relations of intrinsic and extrinsic factors with DNAm.

### Extrinsic factors

Our framework specifies ways in which to study the relations of race and ethnicity with DNAm in the context of extrinsic environments that are interrelated with the intrinsic elements, especially self-identified race and ethnicity. Extrinsic factors also have dynamic relations with other intrinsic factors, such as sex and age. Hence, the environment and experience are expected to change over the life course, and to differ across developmental stages and in relation to an individual’s sex. We highlight early-life environmental factors that may be culturally regulated, which aligns with the DOHaD perspective. Guided by the developmental niche model, we focused on extrinsic factors under three subsystems that operate in concert and interact with each other to form the cultural context of child development: physical and social settings in which the individual lives; culturally regulated customs, such as caregiving and childbearing practices; and caretaker’s psychology, such as feelings, goals, and beliefs [60]. Integrating these three subsystems in our framework, we identified environmental factors for which there is already some, albeit primarily correlational, evidence of their associations with DNAm that can be extended to the context of race and ethnicity, and underscored structural factors relevant to each system.

## Current state of DNAm research in the context of race, ethnicity, and health

Here, we delineate how the intrinsic and extrinsic factors in the framework contribute to our understanding of DNAm and how they have been studied in the context of race and ethnicity. To illustrate each aspect of the integrative framework, we present an overview of the current state of DNAm research in race, ethnicity, and health using 49 studies that were carefully screened and selected to meet criteria of addressing racial and ethnic differences in human health and DNAm in a recent review paper [6]. In their original analyses of this set of studies, the authors took a critical view and evaluated whether these studies “implicitly reflect or reiterate some aspects of the environmentally driven template” (p. 10) [6]. In light of their longer historiographic premise and historical review, they identified a list of current issues, including the focus on negative exposure and pathology, limited research on reversibility of early-life effects, and racial typologies without considering heterogeneity within populations [6]. This previous paper provided a cautionary review of how epigenetic research could be misused to propagate stigmas and presented recommendations for a balanced approach to treat race in postgenomic-era research, including moving beyond using White as the norm and embracing plasticity, variation within populations, and reversibility in epigenetics research [6]. While the current review shared a similar goal of contributing to responsible practices of studying epigenetics in the context of race and ethnicity and examined the same set of papers as in the previous review, it made a distinct contribution by extending the discussion to evaluation of *methodologies and analyses* used in these studies and provided actionable recommendations for future research based on our evaluation.

Specifically, our review was guided by four questions: (1) Did the authors acknowledge how race and ethnicity were measured and describe what measures were used? (2) Did they measure genetic ancestry, and how was it considered in their analyses? (3) Did the authors empirically examine the effects of genetic variants on DNAm? and (4) Did the authors empirically examine any environmental effects on DNAm, and if so, what measures of environment/experience were considered in the analysis? Full details of each study, including information related to the four questions outlined above, the country of each corresponding author’s research institute, the journal in which the study was published, technology used to measure DNAm and genotype, and age of the participants, can be found in Additional file 1: Table S1.

### Intrinsic factors

#### Racial and ethnic identity

**Fig. 3:**
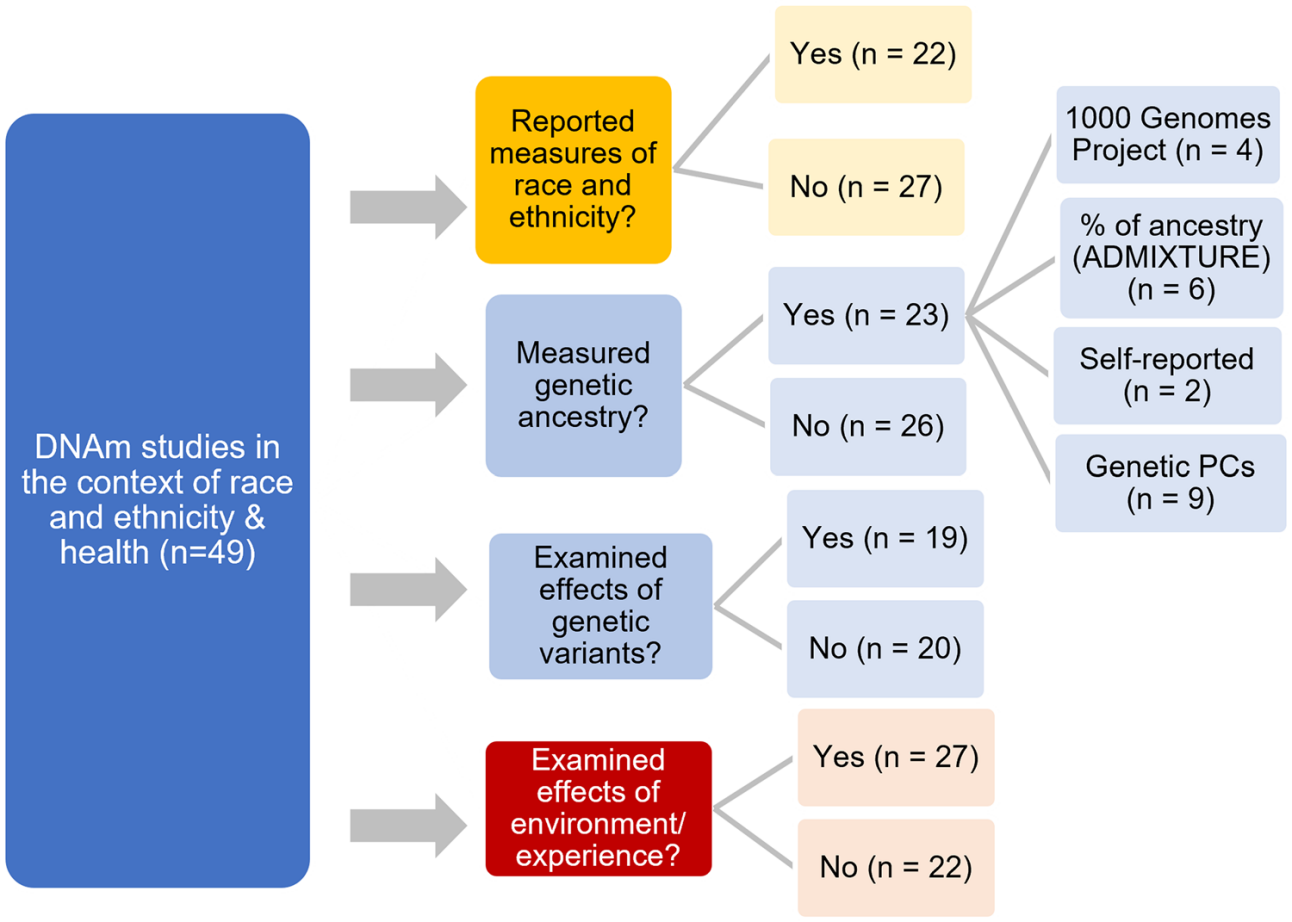
Summary of the 49 studies included in the previous review based on whether they acknowledged how race and ethnicity were measured, whether they measured genetic ancestry and how was it considered in their analyses, whether they empirically examined the effects of genetic variants on DNAm, and whether they empirically examined the effects of environment/experience on DNAm. PCs, principal components

Less than half of the studies reviewed (22 of 49; 44.9%) explicitly discussed how race and ethnicity were measured (Fig. 3). As expected, the majority of those that explicitly reported these measures used self-reported identification (or parent/guardian-reported identification for participants younger than 18 years) [64, 65]. In rare cases, biological signatures were used to categorize participants. For example, one study categorized participants as indigenous Huichol based on the criteria established by the National Institute of Indigenous People of Mexico, as this group has a biological signature of a specific blood type (type O and Rh+) [66]. Most of the studies did not adopt a critical view of race and ethnicity as poor proxies of human physiology [6, 67]. In addition, they did not acknowledge the malleability of racial and ethnic identity over time or based on context and experiences, unlike the genetic architecture [68, 69]. For example, none of the studies addressed or measured the processes of racial or ethnic identity development or sense of belonging [45] as potential influences on experience or biological embedding as measured by DNAm.

#### Genetic ancestry and genetics

The majority of the studies overlooked the effects of genetics on DNAm (Fig. 3). Arguably, however, differences in the underlying genetic backbone onto which methyl groups can be deposited are among the largest contributors to DNAm levels in human differential DNAm studies [24]. There are several potential mechanisms by which this can occur. For example, as DNAm occurs at cytosines in the context of CpG dinucleotides, a polymorphic site with a SNP in which the guanine is replaced by another nucleotide, such as thymine, would not be methylated. In addition, the presence of certain sequence variants before or after CpGs can make methylation at a given site more or less likely. Therefore, the majority of these SNPs strongly associated with CpG methylation levels, known as methylation quantitative trait loci (mQTLs), are located physically close (usually within 50 kb) to the CpGs they influence in the genome [70]. These SNPs close to CpG sites, referred to as *cis*-mQTLs, are thought to disrupt regular protein binding, leading to passive or active changes in DNAm either through steric hindrance with mechanisms of DNAm deposition or directly through interactions with the machinery involved in these mechanisms [71]. These mQTLs were estimated to account for approximately 20%–80% of DNAm variance [72–75], and a significant proportion of commonly characterized DNAm sites on array platforms are likely under genetic influence. For example, a recent report showed that approximately 37.9% of CpGs tested had an associated mQTL in at least one tissue, although many of these were tissue-specific [76]. Notably, genetic influences on DNAm variation were detected among different continental-level ancestries due to differences in the underlying DNA sequences across groups; however, many mQTLs have also been shown to exert their influence on DNAm within populations [70, 77].

Only 38.8% (19 of 49) of the reviewed studies empirically considered the effects of genetic variants on DNAm (Fig. 3). The majority of these studies (16 of 19, 84.2%) identified potential mQTLs in either a post hoc manner following discovery of CpGs associated with the variable of interest in an epigenome-wide association study (EWAS) [78, 79] or by a priori approaches to examine the effects of genetic variants on DNAm [80, 81]. Studies in which the samples lacked corresponding genotyping data leveraged existing mQTL databases (e.g., BIOS QTL browser, NCBI dbSNP database, http://www.mqtldb.org/) [82] or previous meta-analyses of empirical studies [27] to identify the influence of potential mQTLs on CpGs of interest. Alternatively, potential mQTLs were identified by examining the distribution of DNAm level at specific CpGs, as a bimodal or trimodal distribution usually signifies the effects of genetic variants [83]. In other studies, sensitivity analyses were conducted after removing CpGs potentially associated with mQTLs to ensure that the findings were not driven by genetic influences [84, 85] or the effects of SNP mutations [86].

Taking a more holistic view with a data reduction approach rather than focusing on specific SNPs or CpGs, 46.9% (23 of 49) of the studies measured genetic ancestry. The majority of these studies (15 of 23, 65.2%) described genetic ancestry as a continuous variable, with representation of genetic heterogeneity in the sample populations using genetic principal components (PCs) or multidimensional scaling (MDS) coordinates, both commonly employed genomic data dimension reduction techniques. To account for the influence of genetic ancestry on DNAm variation, some studies included the genetic PCs or MDS coordinates in their statistical models [80, 87]. A few studies also acknowledged the admixed nature of the sampled populations and used methods to estimate local and global ancestry with ADMIXTURE [57] and PLINK [79, 85]. On the other hand, some studies used self-reported ancestry based on the continent of origin [88] or self-reported racial identity as a proxy for genetic ancestry [67], which as discussed in previous sections may be inappropriate and could make subsequent meta-analyses across studies difficult.

### Extrinsic factors: Environment and experience

**Fig. 4:**
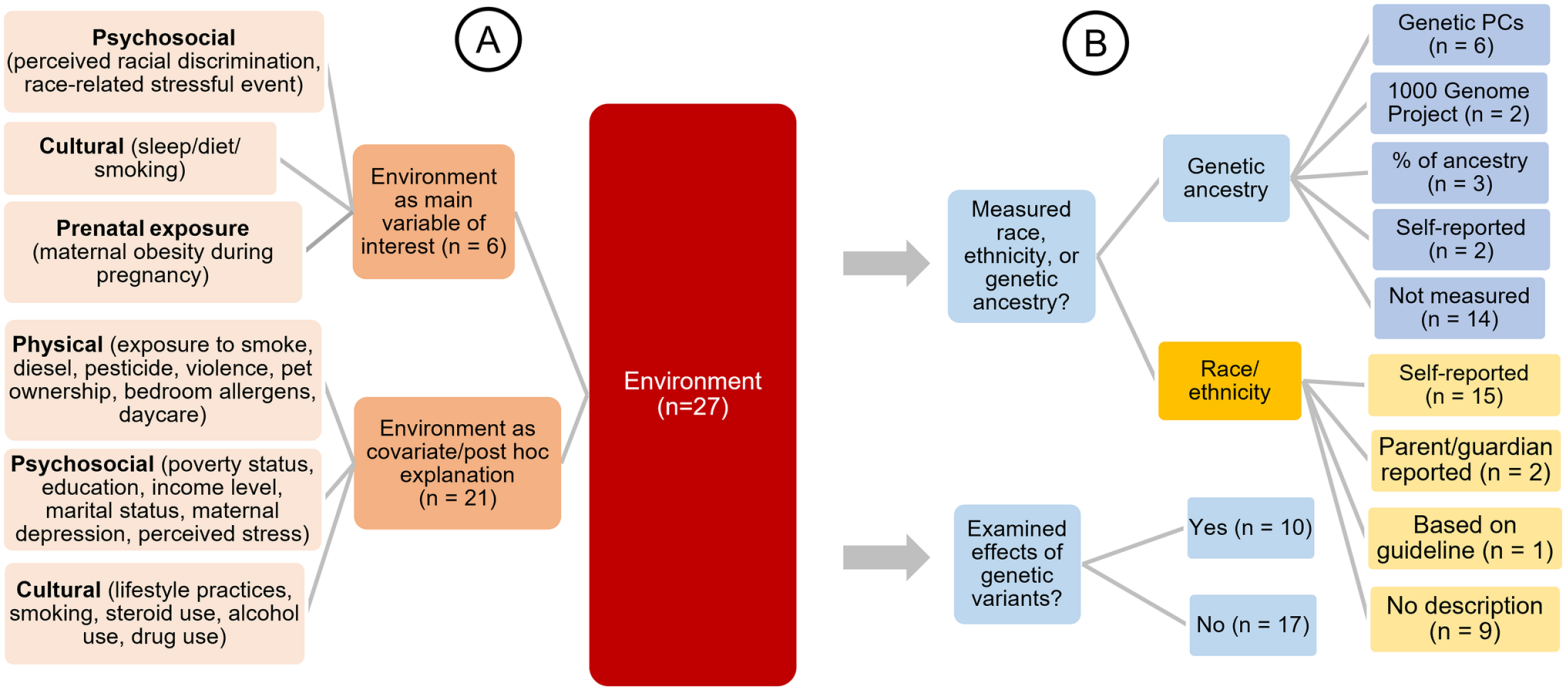
Summary of 27 studies examining environmental factors with **A** categorization of environmental factors as physical, psychosocial, and cultural, and **B** measures of race and ethnicity/genetic ancestry and genetic variant effects

Individuals with different racial and ethnic identities may be exposed to unique developmental niches and sociostructural environments, which can contribute to differential DNAm both within and across race and ethnicity. Although the majority of the studies included in the review discussed environmental associations, most merely speculated on these as potential explanations without empirical investigation, with only 55% (27 of 49) empirically examining environmental exposures or experiences (Fig. 3). Only six of these studies treated an environmental exposure/experience as the main variable of interest [65, 89–93], whereas most included environmental factors as covariates or as post hoc explanations for racial and ethnic differences (Fig. 4a). Furthermore, only two studies explicitly examined whether environmental exposures are indeed different across racial and ethnic groups [67, 94]. Some studies verified whether these identity-associated CpGs were discovered in previous analyses of environmental effects when direct measures of environment were not available for their own samples [95]. We characterized the physical, psychosocial, and cultural aspects of environmental measures relevant to DNAm within the context of race and ethnicity (Fig. 4a), and illustrated how they can become biologically embedded through changes in DNAm and thus have long-lasting impacts on health [96].

#### Physical environment

There is a large body of evidence from correlational data for the association of differential DNAm with physical settings or built environments, including exposure to pollution, chemicals, or toxins, and ecological habitats (e.g., food deserts, availability of green space, neighborhood safety), which can differ across race and ethnicity [97–99]. These physical and built environments (e.g., neighborhoods and households) fall under the subsystem of physical settings, comprising the immediate environment with which children engage in their daily activities. In particular, the physical environments of some minoritized racial and ethnic groups are subject to structural inequity due to social policies, such as redlining, including residing in impoverished neighborhoods with higher likelihoods of exposure to tobacco smoke, allergens, chemicals, and toxins, especially via pollution and occupational hazards, and socioeconomic disadvantages leading to limited resources and poorer nutrition [100]. This is particularly salient in the USA and Canada where racial minorities are exposed to a range of disadvantages resulting from long histories of discrimination and mistreatment [30, 101, 102]. These exposures contribute to health disparities, such as the higher prevalence rates of asthma and allergies among racially minoritized groups [103].

#### Psychosocial environment

In addition to disadvantages within the physical environment, racially minoritized individuals also experience more psychosocial stressors, such as racism, discrimination, and acculturation stress, which shape social settings and experience in their developmental niche [104, 105]. The weathering hypothesis posits that health disparities among racially minoritized groups are consequences of these cumulative daily stressors, which cause “wear and tear” on health throughout the life course [100, 106]. Indeed, perceived racial discrimination and social strain often experienced by African Americans are associated with differential DNAm in genes implicated in inflammation [107] and accelerated epigenetic aging [102, 108], even in cases of outwardly displayed resilience [109]. Hence, beyond self-reported psychological states or behaviors, the underlying impact of stress on the health of racialized and ethnically marginalized groups can be reflected by molecular markers, such as DNAm, although there are methodological limitations to measures of epigenetic aging due to the homogeneity of most DNAm data utilized for developing these tools.

A particularly severe source of psychosocial stress involving race and ethnicity occurs during immigration, especially for those experiencing displacement due to warfare or other sociopolitical crises. Cumulative stressors include, but are not limited to, leaving a familiar environment and social support, becoming a racial and/or ethnic minority in a new society, and the pressure of acculturation, which together represent sources of daily stress that may become biologically embedded. To our knowledge, there have been few studies of the link between differential DNAm and immigration-related stress. Two studies directly examining immigration-related stress demonstrated its association with epigenetic age [110] and differential DNAm at stress-related candidate genes, such as *SLC6A4* [111]. Other studies indirectly addressing this topic compared immigrants with non-immigrants. While some studies echoed the so-called “healthy immigrant” phenomenon referring to a health advantage of immigrants, as indicated by lower epigenetic age acceleration, compared to the native non-migrant population in the home country [112], others did not [84]. These reports showed that whether immigrants do indeed have better health is a nuanced issue, which has also been challenged by a longitudinal study showing convergence of health among immigrants and the native population in the host country after 10–20 years [113]. However, none of the studies we reviewed directly measured perceived immigration or acculturation stress to examine the underlying factors that may explain their findings.

These psychosocial stressors can be associated with the psychology of caregivers, including their beliefs, values, and feelings. For example, African American and Latino mothers, especially those who have experienced racism, are more likely to promote and discuss their children’s racial or ethnic identities than White mothers [114, 115]. Caregiver’s psychology, both pre- and postnatally, including mental health and experience of psychosocial stress, can become biologically embedded in their offspring through DNAm [30, 116]. While most DNAm studies considered maternal factors, such as smoking behaviors, health, and socioeconomic status, few studies have taken into account maternal psychology (e.g., depression) in the context of race and ethnicity [64]. It is also worth noting that very few studies have examined paternal behavioral and psychosocial factors, such as the father’s role in caregiving, their mental health, and their influences on mothers via marital relationships or financial and social support, and their relations with DNAm [117–119]. Furthermore, the effects of race-associated adversity, such as racism and discrimination, are still largely understudied in epigenetics research [30].

#### Cultural environment

Cultural environments are constructed by culturally regulated customs commonly used and accepted by the members of the community of which group members may not even be conscious [60]. Customs are shaped by the psychology of caregivers, including their beliefs concerning their children’s needs and appropriate behaviors, and can be bidirectionally related to the physical and social settings. Cultural practices, particularly lifestyle differences, including drinking and smoking behaviors, sleep, diet, exercise, social networks, and customs surrounding elder care and childbearing and rearing (e.g., breastfeeding, physical touch, and parenting practices), have all been linked to health and development, potentially through adaptations to these cultural environments within physiological systems and the ensuing possibility of biological embedding as measured by DNAm [6, 120–122]. For example, caregiving behaviors, such as breastfeeding, vary considerably across racial and ethnic groups even after adjusting for socioeconomic and psychosocial factors. In particular, mothers from racially or ethnically minoritized groups in the USA who are less acculturated to mainstream practices are more likely to breastfeed [123, 124]. There is substantial evidence in the literature that both the practice and duration of breastfeeding are associated with children’s DNAm of genes implicated in long-term health issues, such as obesity and diabetes [122, 125]. However, additional research regarding other relevant maternal factors related to the effects of breastfeeding, such as diet, is needed. Indeed, diet is another culturally regulated custom that has attracted considerable interest in epigenetics research. The growing field of nutritional epigenetics has demonstrated robust associations of diet quality and nutrition with DNAm, including in dietary clinical trials [126]. Specifically, the “Mediterranean diet,” originating in Southern Italy and characterized by a high proportion of fish, similar to diets commonly found in Japanese culture, has been shown to reduce the rate of cardiovascular disease and to be linked to lower epigenetic age acceleration [127]. Similarly, there is emerging evidence for associations of traditional Indian and Chinese medicine with epigenetic modifications, including DNAm [128, 129]. Thus, the cultural environment comprised of culturally regulated practices and behaviors that vary across individuals with different racial or ethnic identities can contribute to differences in DNAm.

## Considering intrinsic and extrinsic factors together

The relative effects of extrinsic and intrinsic factors can only be disentangled from each other when both are examined empirically. Among the 27 studies included in the review that examined environmental effects, only 10 also empirically investigated the effects of genetic variations on DNAm (Fig. 4b). In particular, two studies examined the associations between differential DNAm and extrinsic factors in conjunction with racial and ethnic identity and genetic effects. Unsurprisingly, both of these studies revealed strong genetic effects such that shared genetic ancestry explained 75% of the variance of DNAm associated with ethnicity [79] and 51% of race-related differentially methylated CpGs were associated with at least one mQTL [64]. However, DNAm of 314 of 916 CpGs associated with self-reported ethnicity remained significantly related to ethnicity even after adjusting for genetic ancestry [79], indicating remaining effects of ethnicity on DNAm that could not be explained by shared genetic ancestry. These studies also examined environmental exposures to shed light on factors capable of explaining differential DNAm associated with race or ethnicity beyond genetic effects. The results showed that some environmental factors known to vary by ethnicity or culture, such as maternal smoking during pregnancy, but not other exposures, such as maternal depression, were correlated with differential DNAm at CpGs whose associations with ethnicity could not be explained by ancestry alone, thus providing a possible environmental pathway for racial and ethnic differences [79]. These studies serve as exemplars to begin to untangle the relative effects of often-confounded genetic and environmental factors on differential DNAm associated with race and ethnicity.

In addition to considering both genetic and environmental effects together, it is also important to examine the interactions between genetics and environment, as recent studies indicated that DNAm at most CpGs can be best explained by gene-by-environment interaction models [23, 130]. Indeed, some studies showed that the relations between DNAm and lifestyle factors, such as cigarette smoking and sleep, varied according to racial and ethnic groups [80, 91]. However, these studies did not discuss their interpretation of these ethnicity-dependent findings and did not report how they accounted for genetic variations across groups. Therefore, it remains unclear whether these findings are a result of genetic effects or cultural specificity.

## Application of the integrative framework in addressing race and ethnicity in future DNAm research

As illustrated by the review of the current state of DNAm studies in the context of race and ethnicity outlined above, limitations remain in the literature on DNAm in addressing racial and ethnic differences and their underlying drivers. Nonetheless, we recognize that social epigenomics is a rapidly growing field and authors of previous studies were likely to have used the most robust and appropriate methods and analyses given the knowledge and technology available at the time. Building on the valuable lessons learned from previous studies and the integrative framework proposed here, we provide recommendations for how to apply the framework and conduct more nuanced and methodologically robust research in future (Table 1).

**Table 1:** Short and long-term recommendations for addressing race and ethnicity in future DNAm research

Short-term recommendations	*Intrinsic factors: Self-identified race and ethnicity* 1. Determine the theoretical and/or biological reasoning for including race and ethnicity in the purpose of the research 2. Clearly define and describe how race and ethnicity are measured and conceptualized, and avoid the interchangeable use of these terms *Intrinsic factor: Genetic ancestry* 3. Describe genetic ancestry along a continuum, and racial derived groupings or ethnicity categories or continent-level ancestry clusters should not be used as proxies for genetic ancestry 4. Account for population structure, specifically genetic heterogeneity, when possible and appropriate based on the goals of the research. This may include stratification, interaction, or adjusting the model for genetic covariates (DNAm-based tools may be used to account for population stratification if genotype data are not available.) *Intrinsic factors: Genetic variations* 5. Examine the effects of genetic variants (Use mQTL databases and/or plot the distribution of CpGs to identify potential mQTLs if genotype data are not available.) 6. Acknowledge limitations of currently available data and tools *Intrinsic & extrinsic factors* 7. Consider interactions between genes and environment as well as between culture and environment 8. Leverage interdisciplinary collaborations and engage local stakeholders in the research process to consider relevant intrinsic and extrinsic factors
Long-term recommendations	*Extrinsic factors* 1. Expand metadata collection to structural and sociocultural factors relevant to race and ethnicity by adopting interdisciplinary perspectives 2. Extend research to examine more associations within the same individual over time, such as in the context of interventions and longitudinal designs 3. Support and fund multidisciplinary and global teams to collect data and interpret results in diverse populations sensitively and appropriately *Intrinsic factors* 4. Improve methodology for characterizing genetic heterogeneity in multiracial and multiethnic individuals 5. Increase diversity of genetic ancestry and racial and ethnic groups in DNAm research and mQTL databases 6. Update DNAm tools to include more genetic, racial, and ethnic diversity in their training and testing sets *Intrinsic and extrinsic factors* 7. Compare individuals with different experiences or environments within the same genetic ancestry, including different ethnic and racial identities

### Application of the proposed integrative framework

The proposed integrative framework can be applied to future research to answer important questions about factors contributing to DNAm variation across racial and ethnic groups in a biologically and culturally sensitive manner. This framework can be used as a guide to which relevant factors should be considered at the stages of conceptualization, research design, data analysis, and interpretation. In particular, the framework illuminates the complex relations between intrinsic and extrinsic factors relevant to both DNAm and race and ethnicity, which should be taken into consideration together to avoid findings that are impossible to interpret and the reaching of inaccurate conclusions. Taking the study of racial disparities in birth outcomes as an example, an overemphasis on genetic differences as explanations may be used to propagate racist narratives. Hence, in addition to genetic variations and other intrinsic factors, it is necessary to investigate how extrinsic factors, such as structural and sociocultural factors (e.g., maternal exposure to racism and diet) [23], may drive some of the biological signals by themselves and/or through their interactions with genetic variations. Specifically with regard to extrinsic factors, our framework extends beyond physical and social factors that are more commonly studied in DNAm research and highlights cultural environmental factors, such as caregiver practices, that may regulate the developmental niches of individuals during early life and contribute to variations in DNAm. In the following sections, we provide actionable recommendations to uncover DNAm signals associated with race and ethnicity, while minimizing the risks of observing signals originating from methodological issues, inherent confounding factors, and/or misinterpretation and inappropriate results.

### Recommendations for addressing race and ethnicity in future DNAm research

In terms of intrinsic factors, most demographic variables, such as sex and age, are commonly collected and can be included in the analysis. However, some factors that should be taken into account, e.g., genetics, may not be available in existing cohorts for which data collection has already been completed, thus posing challenges to good practices in future studies. Despite the argument to withhold investigation of race and ethnicity in the context of DNAm until appropriate data become available, we contend that, as researchers, we have an obligation to fully utilize the valuable biological data provided by our study participants in the most responsible way possible. Here, we present short-term recommendations for handling existing data and suggest possible ways to overcome potential shortcomings inherent in the data, in addition to long-term goals in relation to collecting the types of data, especially those related to extrinsic factors, that will enable more appropriate and culturally sensitive investigation of DNAm in the context of race and ethnicity.

#### Short-term recommendations

The most important constructs among the intrinsic factors in the framework are self-identified race and ethnicity. However, more than half of the studies included in the review outlined above did not describe how race and ethnicity were measured. The first steps to achieve sensitivity and awareness of the complexity of issues related to race and ethnicity are to determine the research goals and how race and ethnicity are involved in the hypothesis [17]. Then, a clearly defined operational definition and clarification of any related terms used, explicitly acknowledging how race and/or ethnicity were measured in the study, and with a critical view regarding what the construct truly represents in a given case are critical for both contextual understanding and interpretation [6, 16, 17, 131, 132].

As discussed above and illustrated in our framework, genetic ancestry and genetic variants are important intrinsic factors closely related to race and ethnicity as well as DNAm, and it is important to account for genetics when possible and appropriate [11]. Furthermore, as the world becomes increasingly diverse and populations are becoming increasingly multiracial, researchers must recognize the complexity of the genetic structures of admixed populations and the increasing importance of describing and analyzing genetic ancestry along a continuous spectrum, both across diverse groups and within seemingly homogeneous groups. In addition, tools are currently available to estimate global fractions of genetic ancestry by individual and, when examining specific areas of the epigenome, local ancestry or the ancestry group from which a particular SNP is most likely to have originated. Together, these approaches provide multiple bioinformatics opportunities to analyze DNAm in the context of genetics in a both sociologically sensitive and biologically accurate manner. Understandably, genotype data may not be available for some cohorts and/or ethnicity information may be incomplete/missing, making it difficult to account for population differences in the study. While not without limitations, a number of DNAm-based tools that allow inference of population structure from DNAm data are available, including EPISTRUCTURE, PlaNET, and Barfield’s SNP-based filtering [44, 134, 135]. In addition to these tools and in the absence of genotyping data, SNP-based DNAm effects can be identified by taking advantage of existing mQTL databases, while acknowledging the limitations of their Eurocentricity. When examining a population or tissue not represented in these databases, it is also possible to use the modality of DNAm (e.g., with the *nmodel* function in the R package ENmix) [136] to identify likely genetic influences, specifically characterizing multimodal CpGs, such as those with a trimodal pattern, indicating DNAm clustered around haplotypes [83]. Finally, CpGs of interest can be compared with those shown previously to be associated with racial and ethnic identity and/or genetic ancestry (see Additional file 2: Table S2 as a resource for future research).

A growing body of research has demonstrated the importance of considering the interactions between genes and environment in studies of DNAm [23], suggesting that individuals with certain genetic backgrounds may have greater susceptibility to some environmental exposures [79]. Similarly, cultural research has also documented social factors, such as perceived social support, which may differentially contribute to an individual’s health depending on their cultural background [137]. Cultural specificity in the effects of environmental exposures/experiences on DNAm can be identified by examining the moderating effects of racial or ethnic identities on DNAm when individuals from different groups are exposed to the same environmental factors [91]. Future research should carefully consider multiple layers of interaction (e.g., gene-by-environment, racial and ethnic identity-by-environment), and examine the relative impacts of genetics and cultural background on an individual’s susceptibility to certain extrinsic factors.

Several statistical frameworks can be applied to address the interconnectedness of these biopsychosocial constructs, such as: model adjustment, which mathematically accounts for the influence of genetic heterogeneity, however it is defined operationally in a given study, on the association between the variable of interest and DNAm [138]; stratification, which can be helpful in the case of distinct groups or otherwise unavoidable confounding factors [139]; mixed models, which are increasingly being used in the genomics space to address population stratification as an issue of kinship, with those of different ancestries being slightly less related than those of similar ancestries, and they may also be applied to DNAm [139]; interaction terms, also known as moderation with hypothesized directionality, can be used to identify CpGs with differing relations to the variable of interest depending on genetic ancestry [80, 91, 140]; and when temporally and theoretically appropriate, mediation models can be adopted to examine whether cultural differences in extrinsic factors, such as customs, have some explanatory power in the associations of race and ethnicity with DNAm, and are widely used in cross-cultural comparisons in psychological research [141]. However, many of these methods have larger statistical power requirements, and it is therefore highly recommended that they be coupled with biologically informed dimension reduction methods to reduce the multiple testing burden of EWASs, such as with the CoMeBack or coMET packages in R [142, 143]. As recommended above, when direct measures of pertinent environments are not available, researchers may also compare their discovered CpGs with race- or ethnicity-related CpGs identified in previous studies of environmental effects on DNAm to infer their relevance [95, 144].

Furthermore, as the study of race and ethnicity in epigenetics requires perspectives from both biological and social sciences, an interdisciplinary team with a range of expertise is necessary. This is also highlighted in other review papers on social epigenomics, including a previous paper reviewing the same set of studies [6, 133]. More importantly, it is crucial to ensure that local stakeholders of the racial and ethnic or cultural communities investigated are involved in the research process to ensure local contexts, values, and goals are preserved, respected, and incorporated into the study [15]. Inclusion of local researchers and other ways to ensure diversity in research teams are nicely described by the *Nature* guidelines on “Authorship: Inclusion and Ethics in Global Research,” which should be used as a reference when planning a study. Taken together, these actions represent important steps to slowly regain the trust of minoritized communities in research.

#### Long-term recommendations

Our framework recommends considering both intrinsic (e.g., genetic) and extrinsic factors while acknowledging the challenge of untangling their effects. One possible solution is to examine individuals within the same genetic ancestry but with a diverse range of experience (e.g., immigration). Some studies compared individuals sharing the same genetic ancestry (i.e., African ancestry) but with different lifestyles and different habitats (e.g., hunter-gatherers or farmers) to assess the degree of intrapopulation variation in DNAm [84, 145]. Such study designs can help to decipher the relative contributions of racial and ethnic disparities in health outcomes [135]. To disentangle the impacts of sociostructural factors from genetic ancestry on DNAm, future studies should compare DNAm of individuals with the same genetic ancestry but different experience, for example, comparison of individuals of African ancestry residing in the USA with those in Africa and with fewer socioeconomic disadvantages.

To avoid the propagation of harmful stereotypes and unwanted misinterpretation of disparities as genetically determined, a more nuanced understanding of structural and sociocultural factors underlying racial and ethnic differences is required. Hence, researchers should expand metadata collection to include environmental exposures and experience relevant to race and ethnicity as denoted by our framework. Only about half of the studies reviewed here examined environmental factors, with very few focusing primarily on environmental effects. As suggested in previous reviews, further investigations of the associations of DNAm with stress and adversity associated with race, such as racism especially during prenatal exposure, are needed [6, 146]. Additional efforts are also required to develop and validate measures capable of capturing important experiences related to race and ethnicity, such as racial trauma and acculturation stress [100, 133].

Interdisciplinary teams consisting of experts in various fields in the biological and social sciences are more likely to have the expertise necessary to identify relevant sociocultural measures that should be included in data collection in studies of DNAm. Furthermore, substantive knowledge across disciplines can ensure that relevant questions and appropriate analyses are used to answer questions related to how sociocultural and structural environments contribute to DNAm in the context of race and ethnicity [133, 147].

It is important to acknowledge that most of the current empirical evidence on the relations between human DNAm and the sociocultural environment related to race and ethnicity remains correlational and does not imply causation. Although we have primarily discussed the impacts of environment and experience on DNAm, we cannot exclude the possibility of bidirectional relations between these factors. Experiments with animal models have yielded some evidence supporting the effects of the social environment, such as maternal caregiving, on DNAm [148], while similar experiments remain impossible in humans for ethical reasons. Nonetheless, there is growing interest in studying DNAm in the context of interventions, and in performing longitudinal studies capable of illuminating how changes in social conditions and environment/experience may be linked to changes in DNAm over time. Specifically, examinations of the longitudinal associations of DNAm with developmental changes in racial and/or ethnic identity may elucidate some of these gene-by-environment effects within the psychosocial perspective. Longitudinal research guided by positive strengths-based approaches can identify changeable extrinsic factors, which can potentially help buffer the negative effects of race-associated stress and adversity on health and provide insight into the associated DNAm processes [100].

In addition, steps should be taken to increase the diversity within both samples and research teams in DNAm research [149]. The previous review reported that the majority of studies on DNAm related to race and ethnicity are still led by researchers based in the USA (77%) [6], including primarily White researchers, and available epigenetic data, both published and biobanked, are largely of populations of European ancestry [149]. The scientific community as a whole should endeavor to support diversity in both the participants entrusting us with their data and in the researchers designing and interpreting the studies [133].

Missing diversity in DNAm research has strong implications for the creation of databases, such as mQTL repositories, and the development of DNAm-based molecular tools, such as methylation risk score (MRS), a score reflective of an individual’s epigenetic susceptibility to certain phenotypes based on the methylation state at CpGs previously identified as linked to the phenotype [150], proxy biomarkers, such as the DNAm-based interleukin-6 (IL-6) predictor [151], and the widely used epigenetic clocks [152]. Comparison across 13 epigenetic clocks, such as the two first-generation clocks (Horvath pan-tissue and Hannum clocks) and 11 second-generation clocks, including GrimAge and the newly developed Pace of Aging clock, showed inconsistent relations of epigenetic age acceleration with race and ethnicity [153]. These results may have been partly due to the small numbers of non-White samples in the training sets and different genetic influences on epigenetic profiles across race and ethnic groups. For example, there is a Eurocentric bias in the discovery cohorts (44%–85% European ancestry) for the three most commonly used epigenetic clocks, i.e., the Horvath pan-tissue clock, the Hannum clock, and the PhenoAge clock [152]. As some genetic variants differ in frequency across genetic ancestries, a DNAm tool developed primarily with data of European ancestry may not be applicable to individuals with non-European genetic ancestry. The Horvath pan-tissue clock is known to be more likely to overestimate the age of individuals who identify as Black Americans and underestimate the age of those who identify as Latin Americans, although whether this is a result of genetics, culture, or societal context, or all of these factors together, remains unclear [84, 152]. In addition, a genome-wide association study (GWAS) of the Horvath and Hannum clocks found substantial genetic associations with epigenetic age acceleration predicted by these clocks, indicating a strong likelihood of an influence of genetic heterogeneity [154]. Meanwhile, the DNAm-based IL-6 predictor was created with an exclusively European cohort [151], even though an allele associated with the density of soluble IL-6 receptors and circulating IL-6 levels has been found by admixture mapping to have frequencies of 4% in West Africans and 35% in European Americans [155]. Given the current limitations of epigenetic clocks and their popularity in both research and the public space, it is important to exercise caution and acknowledge their limitations when examining epigenetic age acceleration in racial and ethnic groups that are not typically included in the training sets. Therefore, future research should involve the recruitment and collection of data from groups with more diverse racial and ethnic backgrounds and genetic ancestries, and the creation of more diverse mQTL databases.

## Conclusion

To move toward conceptualization of human biology as social biology, it is crucial for epigenomics research to take into consideration the sociocultural and structural components underlying self-identified racial and ethnic identity in a methodologically robust and culturally sensitive manner. Hence, our framework advocates a holistic and comprehensive perspective to consider both biological and social factors while incorporating different lenses to promote a productive conversation toward future DNAm research related to race and ethnicity. To facilitate culturally sensitive epigenetics research in the context of race and ethnicity, this paper provided a guiding framework to help recognize and identify relevant structural and sociocultural factors that can contribute to DNAm. At the same time, when addressing the social determinants of health through DNAm, a robust investigation of social impact must include analyses taking into account the underlying genetic architecture upon which DNAm is built and the potential interactions between genes and the environment. With the expanding opportunities for cross-disciplinary collaborations and availability of increasing amounts of biosocial data, epigenetics research has tremendous potential to continue to elucidate health disparities across race and ethnicity.

## Supplementary Information

Links to the electronic supplementary material are provided below.

Supplementary file1 (XLSX 26 KB)

Supplementary file2 (XLSX 1699 KB)

## Data Availability

No empirical data or code were used in this study. Information from previous studies reviewed in this paper are included in the Supplementary Material.
